# Use of Sodium Bicarbonate in Cardiac Arrest: Current Guidelines and Literature Review

**DOI:** 10.14740/jocmr2456w

**Published:** 2016-02-27

**Authors:** Dimitrios Velissaris, Vassilios Karamouzos, Charalampos Pierrakos, Ioanna Koniari, Christina Apostolopoulou, Menelaos Karanikolas

**Affiliations:** a Internal Medicine Department, University Hospital of Patras, Greece; b Intensive Care Department, Brugmann University Hospital, Brussels, Belgium; c Cardiology Department, University Hospital of Patras, Greece; d School of Medicine, University of Patras, Greece; e Department of Anesthesiology, Washington University School of Medicine, St. Louis, MO, USA

**Keywords:** Sodium bicarbonate, Cardiac arrest, Resuscitation, Metabolic acidosis

## Abstract

The aim of the review was to summarize the literature over the last 25 years regarding bicarbonate administration in out-of-hospital cardiac arrest. A PubMed search was conducted using the terms “bicarbonates” and “cardiac arrest”, limited to human studies and reviews published in English (or at least with a meaningful abstract in English) in the last 25 years. Clinical and experimental data raised questions regarding the safety and effectiveness of sodium bicarbonate (SB) administration during cardiac arrest. Earlier advanced cardiac life support (ACLS) guidelines recommended routine bicarbonate administration as part of the ACLS algorithm, but recent guidelines no longer recommend its use. The debate in the literature is ongoing, but at the present time, SB administration is only recommended for cardiac arrest related to hypokalemia or overdose of tricyclic antidepressants. Several studies challenge the assumption that bicarbonate administration is beneficial for treatment of acidosis in cardiac arrest. At the present time, there is a trend against using bicarbonates in cardiac arrest, and this trend is supported by guidelines published by professional societies and organizations.

## Introduction

Sodium bicarbonate (SB) administration has been considered an important part of treatment for severe metabolic acidosis in cardiac arrest, because, based on pathophysiologic considerations, normalization of extracellular and intracellular pH was considered a meaningful endpoint of resuscitation. Correction of metabolic acidosis with SB was recommended by early advanced cardiac life support (ACLS) guidelines published in 1976 [1], and SB was the medication most frequently used during cardiac arrest until the mid-1980s [2]. However, because of concerns regarding potential benefit vs. harm, SB use fell progressively to almost no use by 1991, according to one study from the UK [3]. At the present time, SB administration in cardiac arrest is controversial and matter of ongoing debate, and frequency of use varies greatly between medical centers [4]. The 2010 ACLS guidelines for adults published by the American Heart Association (AHA) state that “Routine use of sodium bicarbonate is not recommended for patients in cardiac arrest” (class III recommendation, based on level of evidence (LOE) B) [5], and these guidelines were not reviewed or revised in the last update published in 2015 [6]. However, ACLS guidelines recommend administration of SB 1 mL/kg boluses as needed for hemodynamic stability (adequate mean arterial blood pressure) and QRS narrowing in cases of severe cardiotoxicity or cardiac arrest from hyperkalemia or tricyclic antidepressant overdose (class IIb recommendation, LOE C) [7], and this recommendation was not reviewed or revised in the last revision of the ACLS guidelines published in 2015 [8]. The aim of this review was to summarize the literature of the last 25 years regarding the potential benefit or harm of SB administration for treatment of acidosis in patients with cardiac arrest.

## Literature Search Methods

We conducted a PubMed database search using the terms “bicarbonate” and “cardiac arrest” in the “Title” field. The search was conducted in March 2015, but was updated in November 2015, in order to include the latest update of the AHA guidelines for cardiopulmonary resuscitation. The search was limited to articles and reviews written in English, or articles written in other languages but accompanied by a detailed meaningful abstract in English, that were published in the last 25 years. The bibliography from all extracted manuscripts was further reviewed for identification of additional relevant references which were included in this review.

## Literature Search Results

A retrospective study published by Roberts et al in 1990 attempted to identify predictors of mortality in patients resuscitated from cardiac arrest, and showed that survival was only 4.2% (10 of 238 patients) when SB was used vs. 27.8% (20 of 72 patients) when SB was not used, and this difference was significant (P = 0.049). However, the authors pointed out that poor survival in patients who received SB could reflect more severe illness with presence of severe metabolic acidosis among patients who required SB [9].

An observational cohort study published by Stiell et al in 1995 evaluated 529 patients who suffered cardiac arrest inside or outside the hospital and received epinephrine according to ACLS protocol guidelines. The study was conducted over a 2-year period and used univariate and multivariate logistic regression to assess the association between six ACLS drugs and survival at 1 h and at hospital discharge. With the exception of procainamide, all standard ACLS drugs, including SB, did not have significant association with survival. However, the authors noted that timing of drug administration could be an important factor [10].

A prospective, randomized, double-blind trial published by Dybvik et al in 1995 enrolled 502 adults who were resuscitated after out-of-hospital cardiac arrest due to asystole or ventricular fibrillation and failed the first defibrillation attempt. The study compared outcomes in 245 patients who received 250 mL of SB-trometamol-phosphate mixture vs. 257 patients who received 250 mL of 0.9% saline during resuscitation conducted in accordance with ACLS guidelines, and showed that, although patients resuscitated after out-of-hospital cardiac arrest had metabolic acidosis, buffer therapy did not improve outcome [11].

The benefit of medications recommended by ACLS support guidelines was questioned by a prospective cohort study on 773 patients resuscitated after cardiac arrest published by van Walraven et al in 1998. This study showed that only 269 of 773 patients survived the first hour. Multivariate logistic regression was used to calculate odds ratios (ORs) and 95% confidence intervals (CIs) and showed significant association between unsuccessful resuscitation and use of epinephrine (OR: 0.08; 95% CI: 0.04 - 0.14), atropine (OR: 0.24; 95% CI: 0.17 - 0.35), bicarbonate (OR: 0.31; 95% CI: 0.21 - 0.44), calcium (OR: 0.32; 95% CI: 0.18 - 0.55), and lidocaine (OR: 0.48; 95% CI: 0.33 - 0.71). Despite the observed association between bicarbonate use and unsuccessful outcome, the authors concluded that their study did not find association between ACLS medications, including bicarbonate and successful resuscitation [12].

A review article published by Adgey and Johnston in 1999 assessed earlier publications and concluded that use of buffer solutions should be limited to cardiac arrests where there is documented severe acidosis, and should be given blindly only after prolonged resuscitation, or in cardiac arrest associated with hyperkalemia or tricyclic antidepressant overdose [13].

Similarly, a review published by Datta et al in 1999 assessed basic life support, advanced life support and post-resuscitation care, and concluded that routine use of bicarbonates was not recommended in cardiac arrest [14].

A large retrospective study published by Bar-Joseph et al in 2002 based on the brain resuscitation clinical trial III, reviewed records from 2,915 patients and found a linear relationship between duration of ACLS and bicarbonate use. The brain resuscitation clinical trial III was a multicenter randomized trial comparing standard vs. high-dose epinephrine during cardiopulmonary resuscitation (CPR), while SB use was optional. The authors concluded that, when bicarbonate was used, it was probably used late, and suggested that, because of development of severe metabolic acidosis, bicarbonate administration should start early [15]. However, these findings were contradicted by a second retrospective study published by Bar-Joseph et al in 2005, which was also based on the brain resuscitation clinical trial III database, except it only included patients with out-of-hospital cardiac arrest where the time from collapse to initiation of ACLS was shorter than 30 min, and included data from 2,122 patients. The study groups included patients to whom SB was administered in less than 50% of CPRs and first epinephrine to spontaneous breathing time exceeded 10 min, and groups where SB was administered in over 50% of CPRs and first epinephrine to spontaneous breathing time was < 10 min. Multivariate regression analysis in this dataset showed that earlier and more frequent use of SB was related to improved chance of return of spontaneous circulation (ROSC) and better long-term outcome [4].

Vukmir and Katz published in 2006 the results of a prospective randomized, double-blinded pre-hospital clinical trial that was conducted in Pennsylvania between 1994 and 1998, before early defibrillation by first responders was introduced to clinical practice. Of 874 registered potentially eligible patients with pre-hospital cardiopulmonary arrest, 792 patients were enrolled in the study. The primary outcome was ROSC or arrival to the emergency department with a pulse. The experimental group (420 patients) received 1 mEq/kg of SB after standard ACLS interventions, whereas the control group (372 patients) received equal volume of normal saline. Overall, there was no difference in survival, as 58 of 420 patients (13.9%) survived in the bicarbonate group, compared to 52 of 320 (13.8%) in the control group. However, in the subgroup of patients with prolonged (longer than 15 min) pre-hospital cardiac arrest, survival was 32.8% in the bicarbonate group vs. 15.4% in the control group, and the difference was highly significant (P = 0.007) [16].

A review article published by Spohr et al in 2008 concluded that only a few drugs conferred a proven benefit for short-term survival after cardiac arrest, and suggested that bicarbonates should only be administered during CPR if indicated based on arterial blood gas analysis or in cases of prolonged unsuccessful resuscitation [17].

A retrospective study published by Geraci et al in 2009 reviewed medical records of 88 patients who received SB during cardiac arrest and showed that 27 of 88 patients (31%) received SB without arterial blood gas (ABG) data. In patients where ABG data were available, bicarbonate administration was linked to alkalemia in 16% (10 of 61) of patients. The authors suggested that early ABG analysis in cardiac arrest may help optimize pH and reduce the frequency of empiric, not warranted, bicarbonate use [18].

A review from Lee in 2011 mentioned the role of drugs administration after effective CPR and defibrillation in the cardiac arrest, but based on randomized trials, no drugs or combination of them have shown benefit on long-term survival [19].

A review by Williamson et al in 2012 reported that although short-term outcomes after CPR have improved as result of the administration of code drugs, in most cases there was no significant benefit with regard to the final outcome [20].

A retrospective cohort study published by Weng et al in 2013 included data from 92 patients who presented to the emergency department with cardiac arrest. The authors compared 30 patients who received vs. 62 patients who did not receive SB, in an attempt to assess the effect of bicarbonate administration after prolonged (> 15 min) CPR. Although patients who received bicarbonate had higher percentage of ROSC, regression analysis showed that bicarbonate administration did not significantly improve the rate of ROSC in out-of-hospital cardiac arrest [21]. The findings of all the above clinical studies are summarized in Table 1 [4, 9-12, 15, 16, 18, 21-28]. Studies in the table are listed in alphabetical order based on the first author’s last name.

**Table 1:** Clinical Studies Evaluating the Effect of Sodium Bicarbonate in Cardiac Arrest

Author	Origin, year	Study design	Findings
Aufderheide et al [24]	Wisconsin, USA, 1992	Retrospective chart review, 619 arrest pts, 273 had ROSC	No association between SB and survival
Bar-Joseph et al [15]	Pittsburgh, PA, USA, 2002	Retrospective study, 2,915 pts from brain resuscitation clinical trial III dataset	SB given in 54% of cases, use increased with ACLS duration. SB should probably be given earlier.
Bar-Joseph et al [4]	Pittsburgh, PA, USA, 2005	Retrospective study, 2,122 pts from brain resuscitation clinical trial III dataset with ACLS lasting < 30 min	Earlier and more frequent use of SB associated with higher resuscitation rates and better long-term outcome
Bishop and Weisfeldt [25]	Baltimore, MD, 1976	Experimental data from seven dogs, clinical data from six cardiac arrest pts	SB increases PCO_2_, accentuates intracellular acidosis in poorly ventilated pts, may be useful in well-ventilated pts
Delooz and Lewi [25]	Leuven, Belgium, 1989	Retrospective data analysis	SB > 1 mEq/kg associated with poor outcome
Dybvik et al [11]	Oslo, Norway, 1995	RCT, SB (245 pts) vs. 0.9% NS (257 pts)	SB therapy had no effect on outcome
Geraci et al [18]	Jacksonville, FL, USA, 2009	Retrospective chart review, all CPR cases in 2005 - 2006, 88 pts received SB	SB linked with alkalemia in 16% of pts, recommendation for early collection of ABG sample
Mattar et al [22]	Los Angeles, CA, USA, 1974	Case series, 12 pts, SB in cardiac arrest	Plasma osmolality > 400 mOsm/kg, serum Na concentrations > 200 mEq/L
Roberts et al [9]	Winnipeg, Manitoba, Canada, 1990	Retrospective study, 326 pts	Survival 4.2% (10/238) when SB given vs. 27.8% (20/72) when SB not given (P = 0.049) but SB use may reflect presence of severe acidosis
Stiell et al [10]	Ottawa, ON, Canada, 1995	Observational cohort study, 529 pts in 2 years received epi per ACLS guidelines	Logistic regression did not show association between SB and survival
Suljaga-Pechtel et al [27]	New York, NY, USA, 1984	Prospective observational study, 277 arrests in 226 pts	Survival lower in pts who needed SB, likely due to illness severity
van Walraven [12]	Ottawa, ON, Canada, 1998	Prospective cohort study, 773 pts with cardiac arrest, logistic regression for OR and 95% CI	269 of 773 pts survived the first hour. SB use significantly associated with unsuccessful resuscitation
Vukmir and Katz [16]	Pittsburgh, USA, 2006	RCT, 792 patients, SB (420 pts) vs. placebo (372 pts)	Overall survival 13.9% (110/792), no difference between groups. Trend for improved survival with SB in prolonged (> 15 min) arrest
Weaver et al [28]	Seattle, WA, USA, 1990	RCT, lidocaine (n = 106) vs. epi (n = 93); historical controls (n = 132) for SB	Higher survival with SB infusion, which was done before the study started
Weil et al [23]	Chicago, IL, USA, 1985	Cohort study, 105 cardiac arrest pts, all received SB	Survival lower if pH > 7.55 within 10 min of CPR
Weng et al [21]	Taiwan, 2013	Retrospective cohort, 92 pts (30 with vs. 62 without SB)	SB did not improve rate of ROSC in prolonged (> 15 min) cardiac arrest

ABG: arterial blood gas; ACLS: advanced cardiac life support; CI: confidence interval; CPR: cardiopulmonary resuscitation; min: minutes; epi: epinephrine; pts: patients; OR: odds ratio; RCT: randomized controlled trial; ROSC: return of spontaneous circulation; SB: sodium bicarbonate.

## Discussion

Debate regarding the potential benefit vs. harm from administration of SB in CPR has been ongoing for decades. Bicarbonate administration was recommended by early ACLS guidelines published in 1976 [1], and this recommendation continued when these guidelines were updated in 1980 [29], but concern about potential harm made bicarbonate use in cardiac arrest increasingly controversial in recent years. As significant acidosis is related to serious adverse systemic effects, bicarbonate administration seems a reasonable intervention to counteract the severe metabolic acidosis caused by hypoxia, poor perfusion and increased lactate production in cardiac arrest, in an attempt to mitigate the adverse effects of acidosis, improve response to exogenously administered catecholamines and increase venous return, thereby improving coronary perfusion pressure [30].

Failure of ventilation and perfusion in cardiac arrest causes severe disruption of homeostasis. Tissue hypoxia from hypoventilation or poor perfusion leads to anaerobic metabolism with reduced adenosine triphosphate (ATP) generation and increased lactic acid accumulation, thereby resulting in metabolic acidosis with plasma pH < 7.20 and increased lactate levels [31, 32]. In addition, respiratory failure with compromised ventilation reduces CO_2_ elimination, resulting in CO_2_ accumulation and respiratory acidosis. Severe combined metabolic and respiratory acidosis and impaired oxygen tissue delivery result in cell damage, as evidenced by cardiac dysfunction from decreased myocardial contractility, hypotension, and renal, hepatic and central nervous system injury that can progress to multi-organ failure. Because of concerns regarding the deleterious effects of acidosis, clinicians have used bicarbonates as buffer to offset the high acid production, in an attempt to help the body restore normal homeostasis in cardiac arrest.

Data published in the 1970s raised concerns that SB administration during cardiac arrest can worsen the outcome after cardiac arrest and emphasized the adverse effects of bicarbonates, including increased osmolality [22]. These concerns were supported by experimental animal studies showing that administration of SB can have adverse effects in dogs with hypoxic lactic acidosis [33], and by clinical data showing that patients with cardiac arrest who received SB in accordance with then current ACLS guidelines had sharply lower survival if pH > 7.55 in the first 10 min after resuscitation started [23]. These arguments were rebutted in a review published in 1987 by Narins and Cohen entitled “Bicarbonate therapy for organic acidosis: the case for its continued use” [34]. However, newer data from experiments in pigs showed that hypertonic buffer solutions in the absence of vasopressors can reduce coronary perfusion pressure below critical thresholds during cardiac arrest and CPR, and may adversely affect outcome [35]. In contrast, an experimental study published by Liu et al in 2002 showed that administration of bicarbonate buffer solution promoted cerebral reperfusion and mitigated cerebral acidosis after restoration of spontaneous circulation in piglets [36]. Similarly, data from experiments with CPR in rats suggested that administration of SB or other buffer solutions can improve survival by ameliorating post-arrest myocardial dysfunction [37]. However, despite encouraging experimental data, concerns about possible detrimental effects of SB administration in cardiac arrest remain.

Few human studies have examined the benefits of bicarbonate administration in cardiac arrest, and most of them are dated before 1990, at a time when SB administration was routine during CPR, even though the acid-base status of patients was not known in the majority of cases. However, that practice has changed over the years due to concerns about adverse effects of bicarbonate administration and the fact that published clinical studies failed to show specific benefits from their use [11, 24, 25, 38].

Published data in recent years suggest that SB administration can have deleterious effects during cardiac arrest, including increased intracellular acidosis, reduced cardiac output, shift of the oxygen dissociation curve to the left, with increased affinity of hemoglobin for oxygen resulting in reduced oxygen tissue release, hypernatremia and hyperosmolarity [39]. As increased blood and tissue CO_2_ concentration leads to worsening of tissue acidosis in major organs, including the heart, possibly contributing to cardiac dysfunction, it may be detrimental to cardiac resuscitation [26, 40-43].

In response to concerns raised by these studies, routine use of SB in cardiac arrest has been discouraged, and the AHA has deemphasized its use in the ACLS algorithms [5]. The main goal in cardiac arrest treatment is to intervene as early as possible, with emphasis on early activation of the emergency response system, early initiation of CPR and early defibrillation, in an attempt to improve outcome. Drug administration still has a role in attempts to improve organ perfusion during CPR, facilitate electrical defibrillation, reduce myocardial irritability, terminate malignant ventricular arrhythmias, minimize metabolic derangements and protect the brain from the effects of ischemia [44].

Buffering solutions other than SB have also been used to correct the metabolic acidosis during cardiac arrest. A review published in 1998 by Bjerneroth reported that different alkaline buffers have been used, but have not shown any benefit because of numerous deleterious effects [45]. Tribonat, a mixture of THAM, acetate, SB and phosphate, has been proposed as suitable alternative to conventional buffer solutions. A review published by Bjerneroth in 1999 assessed 76 publications and, although it did not find improvement in overall survival, it suggested that Tribonat may be superior compared to previously used buffer solutions in cases where administration of an alkalinizing agent is indicated [46].

Some studies have suggested a beneficial role for bicarbonate in the treatment of metabolic acidosis associated with cardiac arrest of prolonged duration, while other studies showed that bicarbonate administration may be counter-productive because it increases tissue and central venous blood carbon dioxide tension. Decisions regarding bicarbonate administration should therefore be based on central venous blood gas analysis [47]. Historically, a report by Stewart in 1964 described 12 cases of cardiac arrest and, although it showed that correction of metabolic acidosis increased the chances of successful treatment, it also suggested that bicarbonate administration is not warranted in all cardiac arrest cases [48]. A retrospective study published by Aufderheide et al in 1992 reviewed data from 3 years of clinical experience with use of SB in 273 patients successfully resuscitated from cardiac arrest, and assessed the potential harms from bicarbonate use. The Aufderheide study showed that 2% (four of 215) of patients who received SB had hypernatremia, but there was no difference with regard to alkalosis, and because of study design limitations, the authors could not determine the influence of bicarbonate use on survival [24]. Also a review by Vukmir et al published in 1996 analyzed all studies before that time showing that six of nine studies reported some prolongation of survival time after cardiac arrest, with one study reporting worse outcome and two studies showing no effect of bicarbonate on survival, but most of these studies were uncontrolled [30]. A brief review published by Professor Adgey in “Heart” in 1998 suggested that, based on available evidence, the mainstay for maintenance of acid-base balance in cardiac arrest is adequate alveolar ventilation. The review recommended that treatment with SB should be reserved for cardiac arrest patients in one of the following four groups: 1) severe acidosis with arterial pH < 7.1 and base excess < 10), 2) patients with prolonged (> 10 - 20 min) cardiac arrest, 3) cardiac arrest related to hyperkalemia, or 4) cardiac arrest related to overdose of tricyclic antidepressants [39].

Although SB use has been part of standard therapy for treatment of acidosis in cardiac arrest, data published over the last 25 years do not support its use. The AHA revised the ACLS guidelines since the 2000 edition of standards and guidelines for CPR and emergency cardiac care (ECC), so that SB administration is only advised at the discretion of the physician directing the resuscitation. In cases where clinicians choose to administer bicarbonates, SB should be given as IV bolus or by IV infusion, with standard dose being 1 mg/kg of body weight as initial dose, followed by 0.5 mg/kg every 10 min for the duration of the arrest. A 50-mL bolus of SB will raise serum pH approximately 0.1 of a pH unit [7]. The 2010 revised AHA guidelines for CPR and ECC emphasize that acidosis and acidemia are dynamic processes resulting from the absence of blood flow in cardiac arrest [5], therefore high quality CPR and early defibrillation in attempt to restore spontaneous circulation are the best methods to restore acid-base balance, with additional benefit gained by ventilation. The majority of studies cited in these guidelines showed poor outcomes and no benefit from bicarbonate administration, while only two studies demonstrated increased ROSC and survival to hospital discharge. In addition, the AHA 2010 ACLS guidelines mentioned significant potential adverse effects related to bicarbonate administration during cardiac arrest, including inactivation of simultaneously administered catecholamines, reduction of systemic vascular resistance, hypernatremia, hyperosmolality and extracellular alkalosis despite intracellular PCO_2_ excess [7].

Because of the above reservations, current ACLS guidelines recommend bicarbonate administration only in cases of cardiac arrest related to hyperkalemia or tricyclic antidepressant overdose [7]. Clinicians can repeat the bicarbonate dose according to patients’ clinical status or based on results of blood gas analysis. Alternative, non-CO_2_ generating buffers, such as THAM and Tribonate, have potential for minimizing the adverse effects of SB, but clinical experience and outcome data are very limited.

Last, according to the ACLS guidelines published by the AHA, routine SB administration is not recommended in the ACLS protocol for pulseless electrical activity, and this is a class III recommendation, based on LOE B (limited populations evaluated, data derived from a single randomized trial or non-randomized studies) [5].

## Conclusion

Although many studies have shown little/no benefit and perhaps harm from administration of SB for rapid correction of acidemia accompanying cardiac arrest, and the latest ACLS guidelines published by the AHA do not recommend routine administration, SB is still used as part of resuscitation in cardiac arrest. Additional research is needed to elucidate further the effects of SB on organ function, on the likelihood of ROSC and on survival in patients resuscitated from cardiac arrest. An objective reappraisal of the use of SB or other buffer agents and perhaps on an appropriate “therapeutic window” for use of SB in cardiac arrest patients is warranted.
